# Unravelling colloid filter cake motions in membrane cleaning procedures

**DOI:** 10.1038/s41598-020-76970-x

**Published:** 2020-11-18

**Authors:** Arne Lüken, John Linkhorst, Robin Fröhlingsdorf, Laura Lippert, Dirk Rommel, Laura De Laporte, Matthias Wessling

**Affiliations:** 1 RWTH Aachen University, AVT - Chemical Process Engineering, Forckenbeckstraße 51, 52074 Aachen, Germany; 2 DWI - Leibniz Institute for Interactive Materials, Forckenbeckstraße 50, 52074 Aachen, Germany; 3 RWTH Aachen University, ITMC – Polymeric Biomaterials, Forckenbeckstraße 50, 52074 Aachen, Germany; 4 RWTH Aachen University, AME – Advanced Materials for Biomedicine, Forckenbeckstraße 55, 52074 Aachen, Germany

**Keywords:** Chemical engineering, Colloids, Gels and hydrogels

## Abstract

The filtration performance of soft colloid suspensions suffers from the agglomeration of the colloids on the membrane surface as filter cakes. Backflushing of fluid through the membrane and cross-flow flushing across the membrane are widely used methods to temporally remove the filter cake and restore the flux through the membrane. However, the phenomena occurring during the recovery of the filtration performance are not yet fully described. In this study, we filtrate poly(N-isopropylacrylamide) microgels and analyze the filter cake in terms of its composition and its dynamic mobility during removal using on-line laser scanning confocal microscopy. First, we observe uniform cake build-up that displays highly ordered and amorphous regions in the cake layer. Second, backflushing removes the cake in coherent pieces and their sizes depend on the previous cake build-up. And third, cross-flow flushing along the cake induces a pattern of longitudinal ridges on the cake surface, which depends on the cross-flow velocity and accelerates cake removal. These observations give insight into soft colloid filter cake arrangement and reveal the cake’s unique behaviour exposed to shear-stress.

## Introduction

Membrane micro- and ultrafiltration is a well established low-energy unit operation for separating and concentrating particles and colloids in the fields of, e.g., food processing, water treatment, and biotechnology. In such filtration processes, a trans-membrane pressure (TMP) drives the fluid through the porous structure of the membrane while larger particles are retained by size exclusion and accumulate on the surface of the membrane. This particle retention causes the generation of a cake layer, which yields an additional pressure loss on top of the membrane’s own pressure loss and thus significantly impacts the process energy requirement. Therefore, depending on the application, cleaning methods such as cross-flow flushing with detergent solutions or backflushing from permeate to feed side are widely applied methods in industry^1,2^. Understanding the origin, behavior, and properties of such cake layers in filtration and cleaning processes is of crucial importance to improving current evidence-based filtration models^3–6^.

Compared to hard particle filtration, filtration of soft particles such as colloids or biological matter results in a compressible cake layer. This compression leads to internal mobility within the cake, which facilitates rearrangement of the particles, resulting in higher packing densities, and forms dense gel layers near the membrane surface^7^. Accordingly, the particle material properties such as size, softness, charge, and ability to interpenetrate mainly influence the cake properties^8,9^. Such properties can be tuned in a reproducible manner using colloidal microgels as a model. Microgels are soft polymeric colloids that are well-studied and easily tunable. Fabricated by precipitation polymerization, microgels offer diameters ranging from less than 100 nm^10^ up to $5\,\upmu {}\hbox {m}$^11^. Microgels are tunable to respond to an external stimulus like temperature, pH, ionic strength, or pressure by changing their size or charge^12^. The properties of densely packed microgel suspensions cover deswelling, deformation, and interpenetration effects and nicely mimics other soft matter applications^13–15^. This flexibility and reproducibility makes the use of microgels in soft matter filtration studies advantageous^16–18^.

Characterization of filter cakes on membrane surfaces follows either visual or non-visual methods. Non-visual methods pose the challenge that calibration experiments need to characterize the origin of the measured value, and appropriate models are required to interpret the data. While hard-sphere filtration can be effectively monitored by TMP and flux measurement^1^ or impedance spectroscopy^19–21^ and modeled accurately^22,23^, non-visual approaches for soft-matter filtration are limited by underlying models. Even though these models have increased in complexity and accuracy in recent years, further experimental verification is needed to cover multi-scale phenomena such as compression, deformation, and rearrangement of the filter cake^24–26^. The on-line visualization of such microscopic events of the filter cake needs adaption of the membrane modules. Hence, a popular method is to retain particles by microfluidic structures to study the phenomena in the cake layer. A glass slide represents one channel wall perpendicular to the membrane such that high-resolution imaging of the filter cake cross-section is possible^3,4,16,27^. Studies, monitoring filtration with real membranes give additional insight into the membrane-cake interface^28^, the cake morphology^29^, and cleaning efficiencies^30^. Nevertheless, high-resolution in-situ microscopy often suffers from insufficient process control resulting in pulsating flows or moving membranes as well as from large focal distances stemming from channel design, reducing the fluorescent signal.

Filter cakes on membrane surfaces are highly concentrated packed beds of particles. When removing a filter cake by flowing tangentially across the cake without permeation, the fluid dynamics are similar to granular beds from the field of geology. Granular bends are well studied related to their fluid dynamic behavior. There are two cases where longitudinal patterns occur on the surface of granular beds during particle transport. The first case is in river beds, where water quickly overflows sand sediments. The fluid dynamic forces rip particles out of their sedimentary position and transport them in a so-called bedload layer on top of the sediment’s surface. This bedload layer is a highly packed suspension of moving particles with a flow-velocity profile ranging from no-movements on the sediment side and fast-movements on the overflown side^31^. In shallow water, instabilities can create a regular longitudinal pattern in the bedload, based on the phenomenon of bedload diffusion. The wavelength of the pattern is larger than the flow depth, such that different flow velocities induce diverse transport rates of the particles. These velocity gradients create instabilities, vortexes in the bedload, and a sinusoidal longitudinal pattern^32^.

In the second case, granular flows, such as sand particles, slide down steep incline planes. There is no overflowing liquid fluid inducing a bedload, but the rapidly moving granular particles collide and form a highly agitated layer. The average density of this layer is smaller compared to the sediment. In such flows, mechanical instabilities can also generate longitudinal patterns, as reported by Forterre and Pouliquen^33,34^ and explained by the concept of granular temperature. The agitation is induced on the overflowed rough bottom, such that the granular temperature at the bottom is highest resulting in a density gradient in the opposite direction than gravity. This density gradient creates vortexes, well known as Rayleigh–Taylor instabilities in fluids. Other experiments and CFD-DEM models confirmed the explanation by showing similar results^35,36^, and a larger scale study questions if this phenomenon might also occur in landslides in the geological context^37^. Transferring the phenomena of longitudinal pattern formation from geology to membrane filter cakes, neither of these has yet been reported.

In this work, we visualize the motions of microgel filter cakes by confocal laser scanning microscopy. The $\sim 2\,\upmu {}\hbox {m}$ sized transparent soft microgels comprise a fluorescently labeled 200 nm core for imaging the center position. We filter them on top of a commercial polyethersulfone (PES) membrane with a pore size of 100 nm and visualize the cake’s top view and cross-section. We show the morphology as well as dynamic bulk motions of the filter cake during two different cleaning procedures: backflushing and cross-flow flushing. Cross-flow flushing unexpectedly creates a longitudinal pattern on the cake surface, similar to the ones obtained in the geological context of granular beds. We analyze the behaviour of this pattern regarding the average cross-flow velocity, its stability, and its influence on cake removal.

## Results and discussion

A custom filtration module with a build-in glass slide parallel to the membrane was fabricated by additive manufacturing and used in all filtration experiments. Poly(N-isopropylacrylamide)-co-acrylic-acid (pNIPAM-co-AAc) microgels were filtrated in two experiment sets by either constant pressure or constant flux filtration. The filter cake was simultaneously monitored by confocal microscopy. In order to monitor the membrane in situ, precise control of the membrane position was necessary. For this purpose, we inserted a permeate spacer structure positioning the membrane surface $700\,\upmu {}\hbox {m}$ above the glass slide in all filtration states. For larger channel size, the fluorescent signal was found to weaken, and microscopical resolution suffered. All filtration experiments were conducted in laminar flow condition with a Reynolds number $2< Re_{Channel} < 250$ for the channel and $0.004< Re_{Particle} < 0.4$ for a particle with a diameter of $2\,\upmu {}\hbox {m}$ as characteristic length. Assuming the feed overflowing the particles with a diameter of $2\,\upmu {}\hbox {m}$ as characteristic length at the filter cake surface with an average cross-flow velocity of ${\bar{u}} > 0.1$ cm/s, we calculated Peclet numbers $\hbox {Pe} > 15000$, which shows that the fluid transport is advection-dominated.

### Cake morphology during filtration

**Figure 1:**
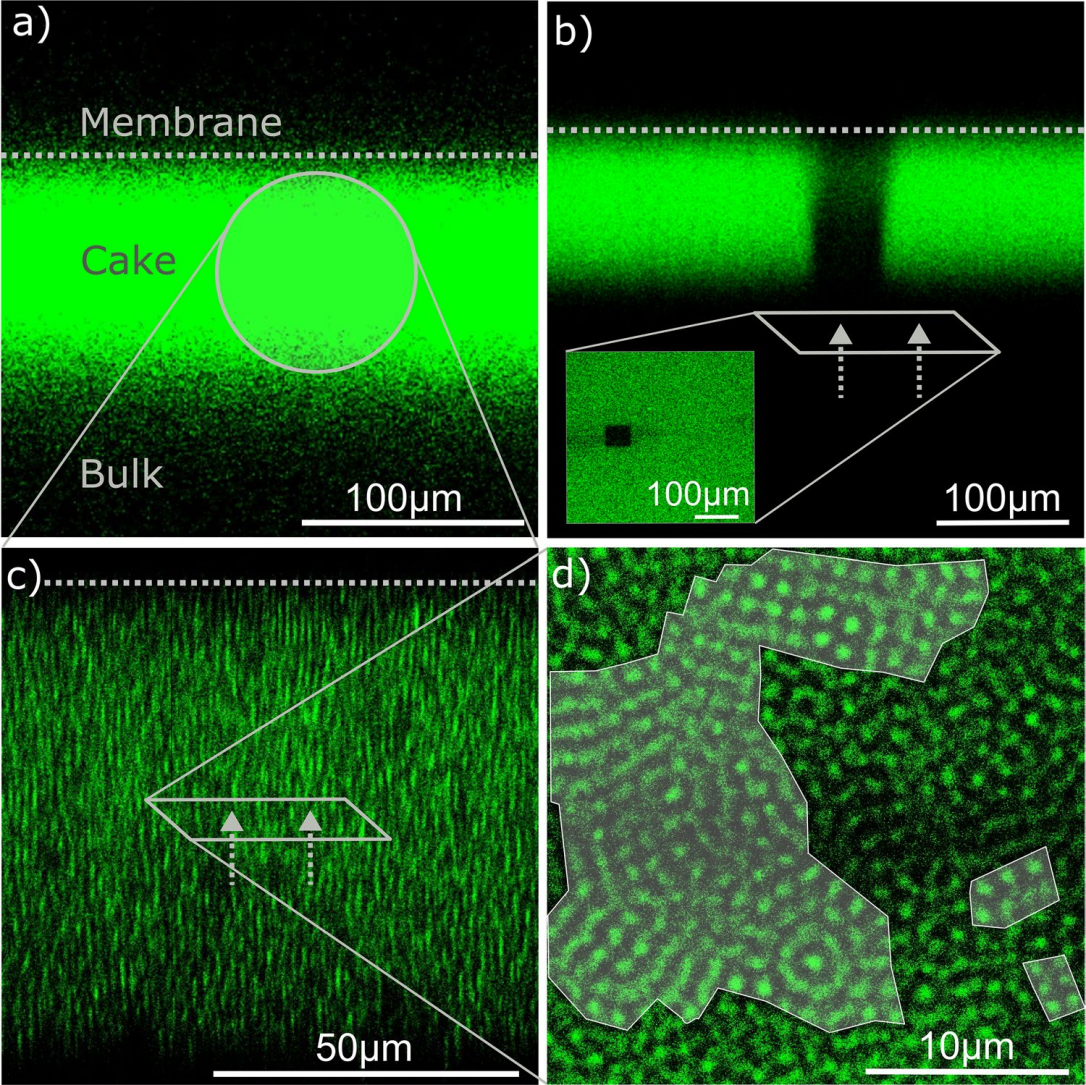
(**a**) On-line filter cake visualization by confocal microscopy in cross-flow constant pressure filtration with a labeled membrane, filter cake, and bulk. (**b**) Filter cake mobility experiment under cross-flow filtration. The image shows the persistent photo-bleached region (black stripe) in cross section and top view (square) not moving during cross-flow filtration. The membrane position is indicated with a grey dashed line. (**c**) Magnification of the cross-section with 63$\times$ lens enables identification of individual microgels. The membrane position is indicated with a grey dashed line. (**d**) A representative slice taken parallel to the membrane, demonstrating the morphology of the filter cake with highly ordered domains highlighted in grey.

Using the custom filtration module, we conducted a constant pressure cross-flow filtration using the labeled microgels (find details in the “Method” section), which deposit as a thick cake layer on top of the membrane surface. Using laser scanning confocal microscopy, we can visualize the top view (x–y-image) and the cross-section (x–z-stack) of the filter cake. Using a 10$\times$ objective, we visualize the shape of the cross section of the filter cake. This method allows an efficient and fast study of the cake’s general shape and its mobility on a large observation field of up to $400\,\upmu {}\hbox {m}$. As shown in Fig. 1a, we first monitored cake growth and identified uniform cake thicknesses all over the membrane. In the laminar flow regime with $\hbox {Re} < 100$, this uniformity is independent of the cross-flow velocity. This cross-flow independency confirms the observations made in traditional filtration experiments^39^.

We studied the macro-mobility of the cake layer conducting a photo-bleaching experiment. For this purpose, we illuminated a small area of the filter cake with high laser intensity. Under these conditions, in this area, the microgels were still present, but the fluorescent signal disappeared (Fig. 1b). In constant pressure cross-flow filtration with 100 mbar TMP and an average cross-flow velocity of 4.2 cm/s, we were not able to see any changes in the bleached region within an long observation time of 5 min. Accordingly during filtration, no macro-mobility of the cake induced by the cross-flow was observable.

For analyzing single microgel behavior, we utilized a 63$\times$ air objective with long working distance to characterize both the cross-section (Fig. 1c) and a representative slice parallel to the membrane inside the cake layer (Fig. 1d). The cross-section shows the cores of the single microgels appearing as elongated stripes, as well known from 3D-image stacks in confocal microscopy using the z-stack technology^40^. The top-view slice is located in the center of the filter cake, i.e., approximately 10 levels of microgels are above and underneath the visualized slice. The image shows areas with highly ordered arrangements of microgels in parallel lines next to each other, indicating crystalline structures. As criterion for being highly ordered, we chose regions where a minimum of three microgels are located on a straight line and two of these lines being parallel to each other. These highly ordered areas are labeled in grey indicating half-ordered and half-amorphous regions. This existence of highly ordered areas confirms microfluidic observations of partly crystalline and partly amorphous cakes for jammed colloids^41,42^. Based on experimental phase diagrams generated with pNIPAM microgels, this transition between amorphous and crystalline structures develops in the transition region from fluid to crystal behavior at effective volume factions $\phi _{eff} \sim 0.56$^43^.

### Cleaning by backflushing

**Figure 2:**
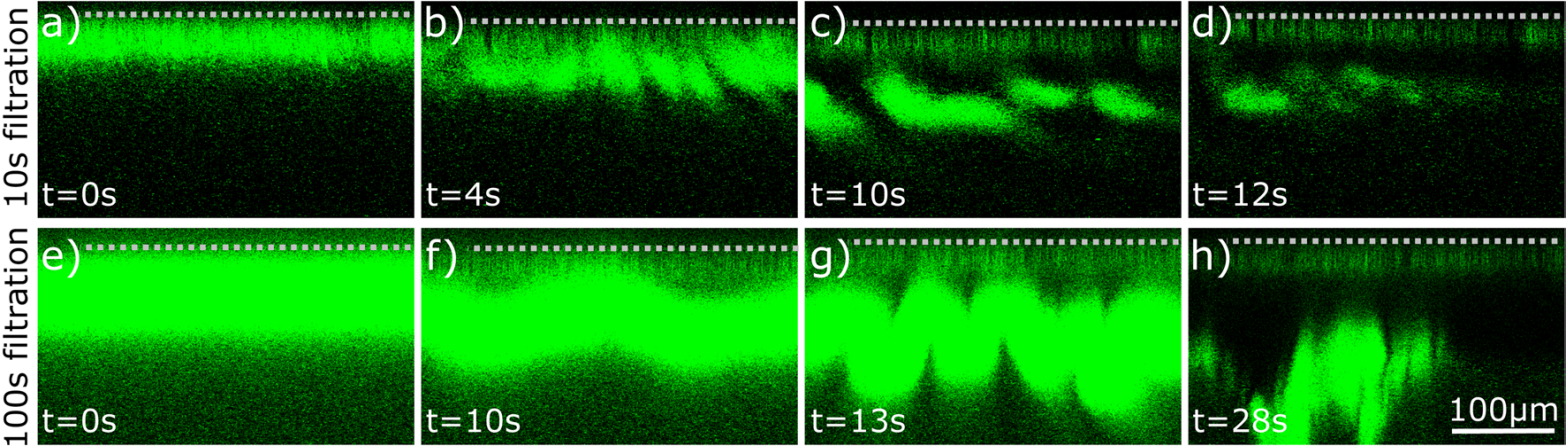
Filter cake backflush mechanism induced by a gentle back pressure of 10 mbar. The membrane position is indicated with the grey dashed line. (**a**–**d**) After 10 s of 300 mbar filtration, the cake detaches in fragmented pieces; (**e**–**h**) after 100 s of 300 mbar filtration, the cake detaches from the membrane in one piece. A monolayer of strongly adhering microgels remains on the membrane and is visible in (**c**,**d**,**g**,**h**). It appears larger due to diffraction of the light.

We additionally studied the morphology of colloidal filter cakes while applying a backflush cleaning procedure. Two sets of experiments were conducted with 10s filtration and 100s filtration, both at 300 mbar constant pressure. After cake built up, we visualized the cake removal by applying a gentle backflush while stopping all other flows. The backflush was induced by a 10 mbar trans membrane pressure on the permeate side, leading to two different phenomena. First, after 10 s of cake build-up, the cake layer detached in fragmented pieces from the membrane and subsequently dissolved in the bulk (Fig. 2a–d, video available in the SI V1). Second, after 100 s of cake build-up, the whole cake layer detached in one piece, bent, and finally broke apart (Fig. 2e–h, video available in the SI V2). At a subsequent cross-flow, hazy fragments were observed in the retentate outlet tube of the module, which subsequently disaggregated in the storage beaker. In both experiments, a shiny layer remains on the membrane surface. At higher magnification, this layer figures out to be a monolayer of microgels adhering to the membrane, appearing broader due to the light’s diffraction at low magnification. The microgels touching the membrane surface deform strongly, such that the area of contact points and subsequently the attractive interaction potential (e.g., Van der Waals forces) increases strongly^44^. Accordingly, the monolayer is hardly removable even at high shear flow rates, as studied by Wiese et al.^28^. These backflushing results lead to the assumption that compression and more time for rearrangement in the filter cake stabilizes the cake structure by increasing the packing density and interpenetration degree. This phenomenon is in accordance with literature, where jamming of microgels has been found to induce entanglement of polymer arms and to increase the adherence to each other^15^. On top of this jamming phenomenon, studies have shown that highly concentrated suspensions tend to rearrange and form crystalline domains during waiting times^45^.

### Cleaning by cross-flow flushing

An alternative way of removing the filter cake from a membrane is cross-flow flushing through the feed channel without membrane permeation. To mimic this procedure, we first filtrated 2.4 ml of a 0.1 g/l microgel suspension in constant flux dead-end mode, ensuring the comparability of the cakes in different experiments. Subsequently, we closed the permeate channel, opened the retentate channel, and applied a cross-flow stream on the feed side of the membrane inducing a shear force on the filter cake surface. For slow cross-flow velocities the cake removal takes several minutes, for high velocities the cake removal only takes a few seconds. While studying these removal kinetics we observed a longitudinal pattern appearing on the filter cake in a certain range of cross-flow velocities. The following sections describe selected properties of this pattern.

#### Pattern formation

**Figure 3:**
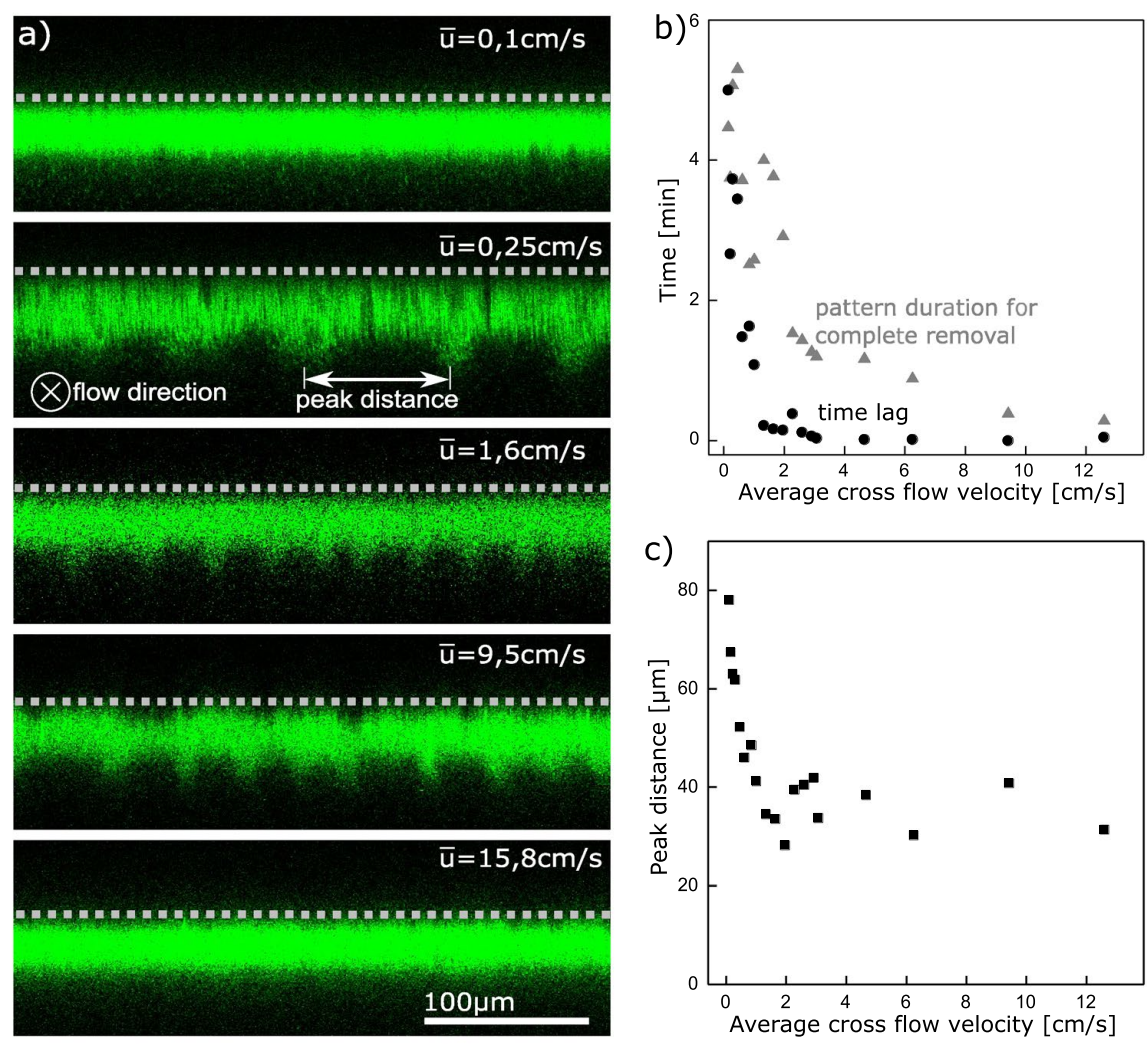
(**a**) Cake removal by cross-flow flushing without permeation reveals a pattern appearing on the microgel filter cake, which depends on the average cross-flow velocity ū. The cross-flow of the bulk is directed into the image-plane parallel to the pattern. The membrane position is indicated with a grey dashed line and the images are taken in the moment of pattern appearance after the time lag. (**b**) The pattern appears with a time lag after starting the cross-flow that depends on the cross-flow velocity (black). The cake removal time after first pattern appearance depends on the cross-flow velocity (grey). (**c**) The peak-to-peak distance in the first moment of pattern appearance depends on the cross-flow only for small velocities.

The pattern has the shape of parallel regular ridges (Fig. 3a) along the flow direction on the whole area of the filter cake covered membrane (Supplementary Fig. S1). During one experiment, unintentionally trapped air bubbles confirmed that the ridges follow the streamlines. The bubbles settled on the feed side of the membrane and the ridges followed the curvy shape around the bubbles, in accordance with the streamlines (Supplementary Fig. S2). The ridges’ appearance and their peak-to-peak distance were found to depend on the cross-flow velocity ${\bar{u}}$ as shown in Fig. 3. For cross-flow velocities higher and lower than the plotted range of $0.2\,\hbox {cm/s}< {\bar{u}} < 13\,\hbox {cm/s}$, no pattern appeared as illustrated in Fig. 3a in the top and bottom images. At low cross-flow velocities in the range from $0.2\,\hbox {cm/s}< {\bar{u}} < 1.5\,\hbox {cm/s}$, a time lag of several minutes occurred before pattern formation was observed (Fig. 3b). Additionally, in this flow range, the peak-to-peak distance at the moment of pattern appearance is larger (Fig. 3c). The ridges stayed on the cake surface for the complete duration of cake removal. They slowly moved in wavy motions, with ridges merging and new ones appearing. The longer the process took, the more ridges merged, such that the peak-to-peak distance during cross-flow flushing increased until the filter cake was completely removed (see supplementary movie V3). The duration for complete cake removal after pattern appearance decreased with higher cross-flow velocity as expected (Fig. 3b).

#### Cake mobility under shear stress

**Figure 4:**
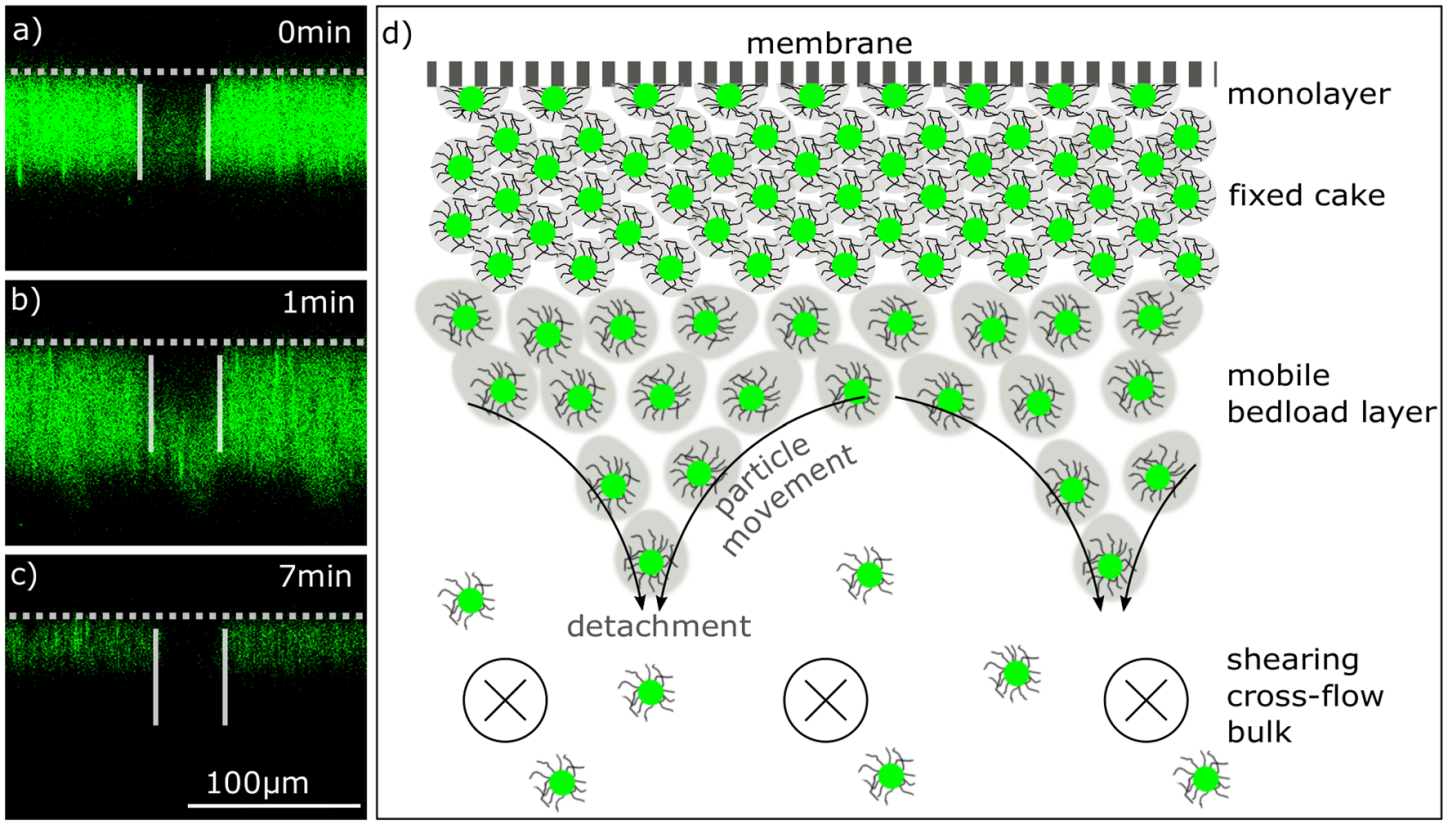
Cake mobility experiment with photo-bleached section. The membrane position is indicated with a grey dashed line. (**a**) After cake formation by dead-end filtration a $40 \times 40\,\upmu {}\hbox {m}$ area of the cake is irradiated with high energy laser light to bleach the microgels. (**b**) Cross-flow flushing without permeation shows a mobility of the cake on the lower part of the surface after the surface pattern appears. (**c**) After complete cake removal, only a irreversible microgel monolayer remains on the membrane. The low resolution makes the single microgels shine fuzzily by diffraction, such that the light cone appears larger than the microgels are. (**d**) The schematic drawing shows the zones of the cake while inducing shear by cross-flow flushing of the bulk: An immobile monolayer on the membrane surface, a fixed cake-zone in the center, and the mobile cake with pattern formation on the bulk-side of the cake. The microgels move from the valley to the ridge, where detachment and dissolution is accelerated.

The cake mobility during pattern appearance was studied by a photo-bleaching experiment similar to the one in Fig. 1. After dead-end cake formation, we exposed a specific region of the cake to high laser intensity and photo-bleached microgels to the extent that they became non-fluorescent despite still being present (Fig. 4a). When inducing the shear stress by cross-flow flushing at 2.4 cm/s without permeation, we observed a fluorescent zone on the bulk side appearing in the bleached region at the very moment of pattern formation (Fig. 4b). In the ongoing experiment the cake layer thickness decreased, but the fluorescent zone remained at the cake-bulk boundary until the complete removal of the cake layer (Fig. 4c). Just as in the backflushing experiments, a monolayer remains on the membrane surface and does not show any movement in the photo-bleached region.

#### Pattern stability at pulsating flow and accelerated cake removal

**Figure 5:**
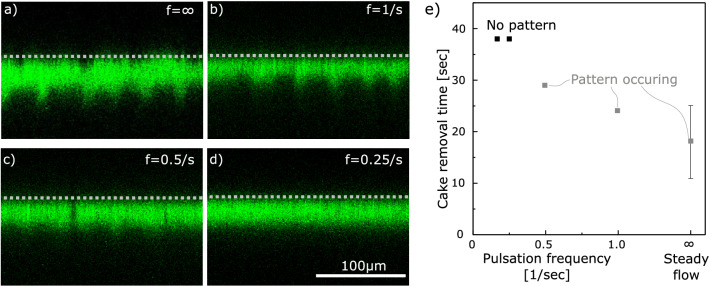
Slowly pulsating flow prevents pattern formation. All images are taken at average cross-flow velocities of ${\bar{u}} = 10$ cm/s and pulsation from 0 to 100 mbar. The membrane position is indicated with a grey dashed line. The pattern appears at no pulsation (**a**) or higher frequencies (**b**), but is reduced (**c**) or inhibited (**d**) at low frequencies. (e) For the equal average cross-flow velocity of ${\bar{u}} = 10$ cm/s the cake removal time increases on the pulsation frequency, while the fastest removal occurs in steady flow. The steady flow error is based on two independent experiments.

We additionally studied pattern stability by pulsating flow, such as it might appear with radial piston pumps or diaphragm pumps. We attached a digital pressure regulator to the feed side and first built up a standardized filter cake by constant pressure dead end filtration. Then we applied a pulsated cross-flow with inlet pressure amplitude from 0 to 100 mbar in frequencies $\hbox {f} = 1$ /s, 0.5 /s, 0.25 /s and 0.17 /s resulting each in the average cross-flow velocity of roughly 10 cm/s. Applying the frequency of $\hbox {f} = 1$ /s (Fig. 5b), pattern formation occurred just as in non-pulsating flow. For $\hbox {f} = 0.5$ /s (Fig. 5c) pattern formation was decreased and for $\hbox {f}\,\le 0.25$ /s no pattern formation was visible. For high frequencies, the soft tubing’s and the module’s damping compensation is dominant, such that pulsation does not inhibit the pattern. For small frequency the damping effect decreases, such that the process does not overcome the lag time that is needed to form a pattern.

Comparing the cake removal kinetics of the pulsation experiments, we find another characteristic of the pattern (Fig. 5e). The steady flow and the high-frequency pulsations with pattern occurrence show a faster cake removal than the slow pulsation frequencies below 0.25/s, where no pattern occurs. Even though this is a limited data-set of six experiments, which does not demonstrate statistical significance, all conducted experiments support the trend. This accelerated dissolution is likely due to the increased microgel mobility in the cake surface, where the pattern occurs. On the one hand, mobility increases the microgels’ distance to each other and hence reduces the inner cake adhesion. On the other hand, the microgels on the tip of the ridge have a larger contact surface to the bulk and experience a higher shear force resulting in detachment. As shown in the scheme of Fig. 4d, particle flow of microgels from the valley to the ridge and subsequent detachment is a possible sequence of the accelerated transport.

#### Discussion of the cake pattern origin

Understanding the origin of the cake movements presented in this study, we look at a single particle in the filter cake in the moment of switching from filtration mode to cross-flow mode without permeation. During permeation, the microgels are compressed by the permeating flux and adhere to each other by the entanglement of polymer chains^15^, hydrogen bonds between NIPAM- and acrylic acid moieties^46^, and hydrophobic interactions between the microgels^47^. When permeation through the cake disappears and shear stress along the flow direction becomes dominant, the microgels on the cake’s surface are ripped out of their fixed position and start flowing parallel to the flow in a bedload layer. Such bedload layers are well known in the field of granular beds (see Introduction). In Fig. 4b, we see that the part of the cake layer next to the bulk shows mobility, while the remaining cake close to the membrane remains fixed. When flowing in a specific cross-flow velocity range (see Fig. 3), the microgels in the bedload start forming the longitudinal pattern due to some instability occurring either inside the cake or by the fluid overflowing the cake. We now discuss three instabilities from other research fields that might promote pattern formation. First bedload diffusion, second vortex formation in a fluid dynamic boundary layer, and third Rayleigh–Taylor instabilities.

As described in the Introduction, bedload diffusion can occur when shallow-water overflows a mobile bedload layer and has a larger wavelength than the water depth. The shallow water leads to different cross-flow velocities at the ridge and the valley, creating different driving forces for particle transport and stabilizing the pattern. In our case, we fulfill the criterion of the mobile bedload layer. Still, the shallow water criterion is not satisfied, as the wavelength (approx. $40\,\upmu {}\hbox {m}$) is smaller than the water depth ($700\,\upmu {}\hbox {m}$), such that velocity differences between ridge and valley are not reasonable.

Another possible explanation for pattern formation is vortex formation in a fluid dynamic boundary layer in the transition range of laminar and turbulent flow. The Tollmien–Schlichting wave is observed at lower Reynolds numbers than in the traditional turbulent transition range. It forms longitudinal vortexes at boundaries by interactions of surface roughness and external perturbations (e.g., acoustic, vortical, temperature, or vibrational fluctuations)^48^. In our case, the more viscous cake and the less viscous bulk might destabilize the boundary layer similar to surface roughness. Still, the second criterion of having an external perturbation is not satisfied. Moreover, fluid dynamic vortexes appear for Reynolds numbers in the range of $\hbox {Re} > 500$^49^, and not for our range of $\hbox {Re} > 2$. Therefore, we do not expect vortex formation in fluid dynamic boundary layers to be a potential mechanism for our phenomenon.

Rayleigh–Taylor instabilities are known to create longitudinal patterns when grains flow down steep inclines. The mean density at the bottom side of the flow decreases, such that Rayleigh–Taylor instabilities create vortexes in the cross-section of the flowing layer (see Introduction). The main criterion for the instability in a density difference in the opposite direction as gravity acts. For meeting the criterion in our case, the higher density bedload layer needs to be positioned above the lower density bulk. Using an inverted microscope, the membrane and the cake are above the feed, meeting the required criterion. Nevertheless, the densities of microgels and the solvent are very similar, such that no settling under gravity is observed, and the influence of gravity is minor. Additionally, we do not observe any pattern without cross-flow stream, suggesting that the mobility in the densely backed bedload layer abolishes adhesive mechanics between microgels and enables gravity-driven movements already at minor density differences. The time lag, which is observed before pattern formation after starting the flow, supports this theory (Fig. 3b). Small cross-flow velocities with reduced shear stress delay the formation of the mobile bedload layer and the subsequent pattern formation.

Finally, we cannot give a definite explanation for the pattern appearance, but the most promising mechanism is a combination of the mobile, less-adherent bedload layer with density-driven Rayleigh–Taylor instabilities. For this mechanism, the pattern would not be specific to soft microgels but to cake layers positioned above the feed channel. Accordingly, we suggest a morphological description with three different layers in the process of pattern formation as shown in Fig. 4d. An immobile monolayer adheres to the membrane surface, while a fixed cake layer is positioned in the center of the cake. At the cake-bulk boundary, a mobile bedload layer is advectively moving along the shear flow direction. In the bedload layer, the mobility and the mean particle distance increases, such that the particle–particle adhesion decreases. Accordingly, small density differences might create Rayleigh–Taylor instabilities and transport the loose microgels out of the bedload towards the bulk, such that a longitudinal pattern occurs. Nevertheless, further research is required to estimate if the microgel’s minor density difference suffices for creating the instabilities.

## Conclusion

This study links general particle specific investigations of densely packed soft colloidal suspensions observed in idealized microfluidic systems to real membrane processes. In this context, we present a unique visualization of colloidal filter cakes during different process conditions in a cross-flow microfiltration module. As an exemplary colloid, we use pNIPAM-co-AAc microgels with a small fluorescent core. Accordingly, the filter cake is transparent, while the core positions in the whole cake can be detected precisely using confocal microscopy. In-situ measurements of the cake morphology on a single colloid level reveal highly ordered and amorphous regions inside the cake. Based on these results, further research is required for a better understanding under which circumstances crystalline and amorphous regions develop in filter cakes and how they develop during filtration processes. Furthermore, we studied the motions of the filter cake when being removed by backflushing. The experiments reveal that the thickness of the filter cake and it’s internal organization influence the stability of the cake, such that the size of the removed cake-pieces varies. Additionally, we see changes in the cake structure when applying a shear induced by cross-flow flushing along the cake without permeating the cake. The top-most particle layers of the cake mobilize and a pattern on the cake surface appears. This pattern occurs with a time lag and has a characteristic peak-to-peak distance, both depending on the cross-flow velocity. The pattern supports colloid transport from cake to bulk and accelerates cake removal accordingly. We assume Rayleigh–Taylor instabilities in the mobile bedload layer as a physical explanation for the pattern phenomenon.

The novel phenomena of dynamic cake mobility presented in this study introduce a new approach of fouling analysis to the field of membrane filtration. We show that soft compressed cakes adapt their properties to their environment during permeation and shear force by changing their organization and mobility. Nowadays, such dynamic processes are barley studied and practically not considered in module design and process engineering but might give some additional guidance towards process optimization and additionally increasing accuracy of filtration models.

## Methods

### Membrane module

**Figure 6:**
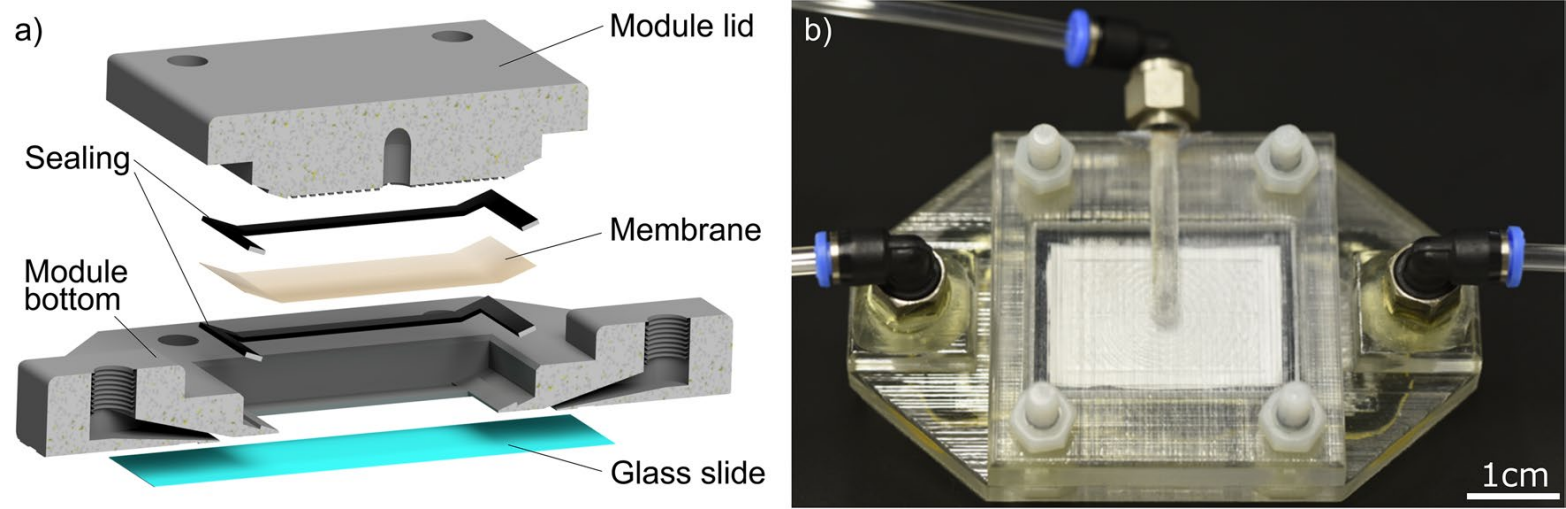
The cross-section model of the module assembly shows the additive manufactured filtration device consisting of a module bottom, a module lid, and the filtration membrane squeezed in between two flat sealing gaskets. The module lid includes a membrane positioning spacer structure and the permeate outlet. The module bottom includes a integrated glass slide, the feed inlet, and the retentate outlet (**a**). The permeate-side view of the real module shows the assembly with four plastic screws, IQS push-in fittings, and tubing (**b**).

A custom membrane module (3D-CAD-model as supplementary information) was designed to enable membrane permeation and in-situ observation of the membrane surface (see Fig. 6). The in-situ observation is implemented by attaching a glass cover slide as module bottom, such that the optical laser microscope can directly observe the membrane surface. Additionally, the feed channel height of $700\,\upmu {}\hbox {m}$ is adjusted by positioning the membrane with an imprinted spacer structure in the module lid. This spacer structure avoids movements of the membrane on the one hand and reduces the feed channel height which shortens the optical laser path and increases microscope resolution on the other hand. The module was liquid-tight tested for pressures up to 400 mbar and used in the experiments at pressures below 300 mbar.

The module was designed by Autodesk Inventor and fabricated by additive manufacturing with a polyjet printer (Objet Eden 260VS) using “Veroclear” material. The printed module was stored in 1M NaOH solution to dissolve the support material, rinsed with water and dried at room temperature for at least 24 h. Afterwards a $175\,\upmu {}\hbox {m}$ thick glass cover was glued to the module using a two-component epoxy glue “UHU-Plus Schnellfest” and baked at $60^\circ \hbox {C}$ for another 24 hours. The module was assembled with a $0.1\,\upmu {}\hbox {m}$ pore size PES membrane with an active membrane size of 15 mm × 24 mm (Pieper Filter GmbH). The membrane was mounted between two 0.5 mm thick flat gasket frames (Viton/FKM 0.5 mm, Lux & Co GmbH). The membranes were wetted beforehand by immersing them in a 50 vol% water, 50 vol% ethanol mixture for at least 24 h. The module top and bottom were pressed together using four plastic M4 screws and nuts. 3 $\times$ 2 mm polyurethane tubes were connected using M5 $\times$3  mm IQS connectors (Landefeld GmbH).

### pNIPAM-co-AAc microgels

Filtrated colloids are poly(N-isopropylacrylamide)-co-acrylic-acid core-shell microgels that were synthesized as described elsewhere^28,50^. In short, the polystyrene cores have a diameter of 200 nm and are labeled with a nile-red fluorescent marker, such that visual observation of the center of the microgel is possible using a fluorescent microscope. The non-fluorescent pNIPAM shell (14 g/l NIPAM, 1 wt% MBA, 5.8 wt% KPS, 26 mol% or 7 mol% AAc—see section Filtration experiments all percentages are relative to the monomer amount) is polymerized around the cores and creates a soft microgel with a diameter of $\sim 2\,\upmu {}\hbox {m}$ and has a negative charge with a zeta potential of $-37$ mV at pH 7 (measured with DLS—ZetaSizer Ultra). Both microgel batches (26 mol% and 7 mol% AAc) had the same size and Zeta-potential. The synthesis solution was purified from linear chains and residuals of other reactants by dialysis (25kDa, SpectraPor 6, CarlRoth) against DI water for one week while changing the DI-water twice a day resulting in pH7. For all experiments the microgel solution was diluted with water to a concentration of 0.1 g/l. The pH was kept constant at pH 7 during all experiments and checked before and after each experiment.

### Experimental setup

The experimental setup uses a feed side syringe pump (Chemyx Fusion 4000) for precise and pulseless flow and a digital backpressure regulator (Elveflow OB1 MK2+) on the retentate module outlet. Accordingly, experiments could be conducted either in constant pressure cross-flow filtration by adjusting a feed flowrate with the syringe pump and setting a backpressure with the pressure regulator or in dead-end constant flux filtration by closing the retentate channel with a valve. The filtration module is mounted on an inverted confocal laser scanning microscope (Leica SP8) using 10$\times$ air and 63$\times$ air objectives, enabling on-line visualization of slices in the top view and the cross-section to the membrane with a frame rate of $\sim 1$ frame per second at a resolution of 512 $\times$ 512 px. The cross-section images were taken by laser scanning in x–z scan mode. Thereby the laser scans in x-direction and the sample simultaneously moves through the focal plane by the galvo controlled z-stage.

### Filtration experiments

Experiments on cake stability and morphology (Fig. 1) were conducted in constant pressure filtration mode by applying a trans-membrane pressure of 100 mbar and an average cross-flow velocity of 4.2 cm/s. The low trans-membrane pressure of 100 mbar was chosen to reduce the influence of cake growth during the analysis. The average cross-flow velocity is calculated by dividing the feed volume flow by the size of the feed channel cross-section, which has a width of 15 mm and a height of $700\,\upmu {}\hbox {m}$. For mobility studies, an $80 \times 80\,\upmu {}\hbox {m}$ area of the cake was photo-bleached by applying maximum laser power for several minutes. For this experiment the first microgel batch with 26 mol% AAc was used.

Back-wash mechanisms (Fig. 2) were studied by first creating a cake layer by constant pressure filtration at 300 mbar for a specific duration and subsequently applying a gentle gravitational permeate back pressure of $\sim 10$ mbar by hand lifting the permeate outlet tube and stopping the feed flow. The experiments were repeated twice showing the same phenomenon, only images from the one experiments with clearer imaging are shown. For this experiment the first microgel batch with 26 mol% AAc was used.

Pattern formation and its morphology at cross-flow flushing (Figs. 3, 4) were studied by first dead-end cake formation (3 min, 0.8 ml/min), then closing the permeate by a valve to avoid any membrane permeation or permeate backflush, and subsequent cross-flow flushing at different velocities without applying any retentate back-pressure. All experiments conducted in the flow-range are plotted in the graph (Fig. 3b,c). For this experiment the second microgel batch with 7 mol% AAc was used.

The experiments on pulsating flow (Fig. 5) were conducted by connecting two independent channels of the digital pressure regulation (Elveflow OB1 MK2+) to the feed and retentate side. We started with constant pressure cross-flow filtration at 300 mbar for 2 min to generate a filter cake and subsequently applied a pulsating cross-flow by applying a sinusoidal pressure on the feed side ranging from 0 to 100 mbar while closing the permeate side by a valve. For this experiment the second microgel batch with 7 mol% AAc was used.

## Supplementary information

Supplementary Information 1.

Supplementary Video 1.

Supplementary Video 2.

Supplementary Video 3.

Supplementary Information 2.

Supplementary Information 3.
