# Supplementary material for: Unravelling colloid filter cake motions in membrane cleaning procedures
Source: Sci Rep. 2020 Nov 18;10:20043. doi: 10.1038/s41598-020-76970-x (PMC7674421; doi:10.1038/s41598-020-76970-x)
Supplement: Supplementary file 1 — Supplementary Information 1. [file 41598_2020_76970_MOESM1_ESM.pdf]

Unravelling colloid filter cake motions in membrane cleaning procedures

Arne Lüken[1], John Linkhorst[1], Robin Fröhlingsdorf[1], Laura Lippert[1], Dirk Rommel[2], Laura De Laporte[2,3,4], and Matthias Wessling[1, 2] *

[1] RWTH Aachen University, AVT.CVT - Chair of Chemical Process Engineering, Forckenbeckstraße. 51, 52074 Aachen, Germany
[2] DWI - Leibniz Institute for Interactive Materials, Forckenbeckstr. 50, 52074 Aachen, Germany
[3] RWTH Aachen University, ITMC - Institute of Technical and Macromolecular Chemistry
[4] RWTH Aachen University, AME - Institute of Applied Medical Engineering
* manuscripts.cvt@avt.rwth-aachen.de

Supplementary Information

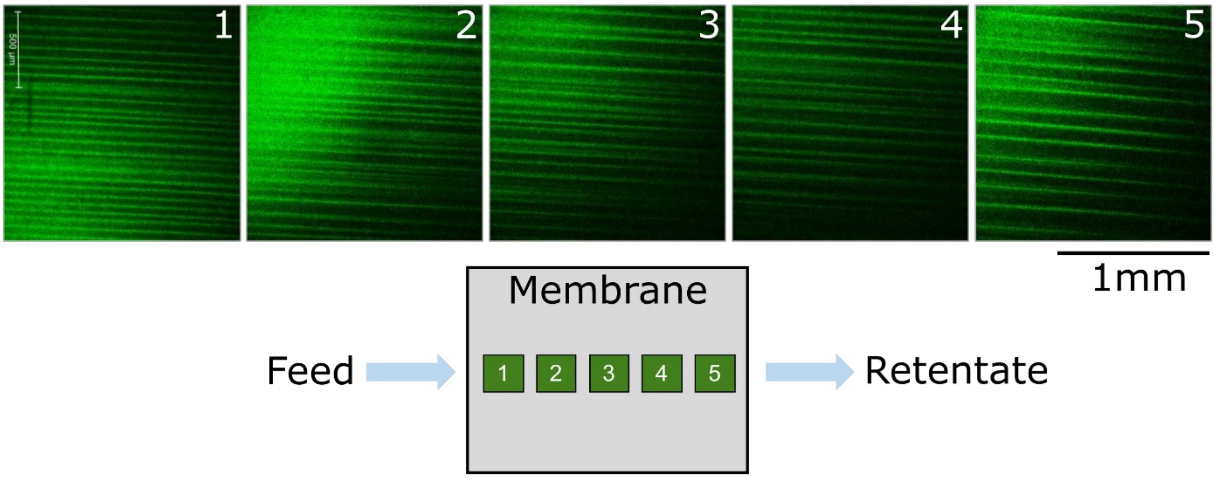

Figure S1: Streaks at different positions on the whole membrane from the feed inlet (1) to the retentate outlet (5) with the flow going from left to right. The peak distance increases slightly from 1-5 because the images were taken one after the other. As can be seen in Supplementary Information V1, the longer the process takes, the more streaks merge.

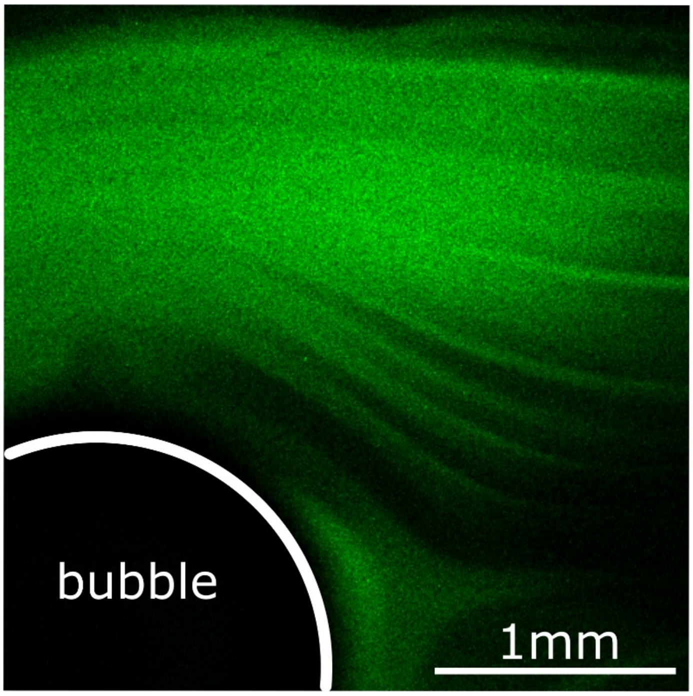

Figure S2: Top view of the pattern while the cross-flow passes an unintentional bubble on the feed side of the membrane flowing from left to right. The streaks merge around the position of the bubble, such as stream lines of the fluid would do.

Video V1: Video of the images recorded during backflushing of a filter cake, that was built up with a constant transmembrane pressure of 300mbar for 10s according to Fig.2 a-d. Compared to the images in Fig. 2 the primary data-video is flipped upside down as it is displayed in the microscope.

Video V2: Video of the images recorded during backflushing of a filter cake, that was built up with a constant transmembrane pressure of 300mbar for 100s according to Fig.2 e-h. Compared to the images in Fig. 2 the primary data-video is flipped upside down as it is displayed in the microscope.

Video V3: Video of the cake removal by cross-flow flushing with a longitudinal pattern occurring on the surface of the filter cake. The real-time is illustrated in the bottom left in the format hh:mm:ss.ms. Compared to the real process and the images in Fig. 3-5 the primary data-video is flipped upside down as it is displayed in the microscope.

3D Filtrationmodule bottom: 3D design file of the filtration module bottom part. For complete assembly, the glass slide is glued to the bottom and feed and retentate tube-connections are screwed in the threads.

3D Filtrationmodule top: 3D design file of the filtration module top part. For complete assembly, a permeate tube-connection is screwed in the thread.
